# Interlaboratory Evaluation of Rodent Pulmonary Responses to Engineered Nanomaterials: The NIEHS Nano GO Consortium

**DOI:** 10.1289/ehp.1205693

**Published:** 2013-05-06

**Authors:** James C. Bonner, Rona M. Silva, Alexia J. Taylor, Jared M. Brown, Susana C. Hilderbrand, Vincent Castranova, Dale Porter, Alison Elder, Günter Oberdörster, Jack R. Harkema, Lori A. Bramble, Terrance J. Kavanagh, Dianne Botta, Andre Nel, Kent E. Pinkerton

**Affiliations:** 1 Department of Environmental and Molecular Toxicology, North Carolina State University, Raleigh, North Carolina, USA; 2 Department of Anatomy, Physiology, and Cell Biology, School of Veterinary Medicine, University of California, Davis, Davis, California, USA; 3 Department of Pharmacology and Toxicology, Brody School of Medicine, East Carolina University, Greenville, North Carolina, USA; 4 Health Effects Laboratory, National Institute for Occupational Safety and Health, Morgantown, West Virginia, USA; 5 Department of Environmental Medicine, University of Rochester, Rochester, New York; 6 Department of Pathobiology and Diagnostic Investigation, Michigan State University, East Lansing, Michigan, USA; 7 Department of Environmental and Occupational Health Sciences, University of Washington, Seattle, Washington, USA; 8 Center for Environmental Implications of Nanotechnology, California Nanosystems Institute, Los Angeles, California, USA

**Keywords:** carbon nanotubes, inflammation, lung, nanoparticles, titanium dioxide

## Abstract

Background: Engineered nanomaterials (ENMs) have potential benefits, but they also present safety concerns for human health. Interlaboratory studies in rodents using standardized protocols are needed to assess ENM toxicity.

Methods: Four laboratories evaluated lung responses in C57BL/6 mice to ENMs delivered by oropharyngeal aspiration (OPA), and three labs evaluated Sprague-Dawley (SD) or Fisher 344 (F344) rats following intratracheal instillation (IT). ENMs tested included three forms of titanium dioxide (TiO_2_) [anatase/rutile spheres (TiO_2_-P25), anatase spheres (TiO_2_-A), and anatase nanobelts (TiO_2_-NBs)] and three forms of multiwalled carbon nanotubes (MWCNTs) [original (O), purified (P), and carboxylic acid “functionalized” (F)]. One day after treatment, bronchoalveolar lavage fluid was collected to determine differential cell counts, lactate dehydrogenase (LDH), and protein. Lungs were fixed for histopathology. Responses were also examined at 7 days (TiO_2_ forms) and 21 days (MWCNTs) after treatment.

Results: TiO_2_-A, TiO_2_-P25, and TiO_2_-NB caused significant neutrophilia in mice at 1 day in three of four labs. TiO_2_-NB caused neutrophilia in rats at 1 day in two of three labs, and TiO_2_-P25 and TiO_2_-A had no significant effect in any of the labs. Inflammation induced by TiO_2_ in mice and rats resolved by day 7. All MWCNT types caused neutrophilia at 1 day in three of four mouse labs and in all rat labs. Three of four labs observed similar histopathology to O-MWCNTs and TiO_2_-NBs in mice.

Conclusions: ENMs produced similar patterns of neutrophilia and pathology in rats and mice. Although interlaboratory variability was found in the degree of neutrophilia caused by the three types of TiO_2_ nanoparticles, similar findings of relative potency for the three types of MWCNTs were found across all laboratories, thus providing greater confidence in these interlaboratory comparisons.

Rapid development of the nanotechnology industry is resulting in the production of a variety of engineered nanomaterials (ENMs) for structural support, electronics, energy, medical imaging, and drug delivery, as well as other applications. Although nanotechnology offers enormous potential societal benefits, concerns have been raised about the safety of ENM-containing products in regard to human and environmental health. Cumulative evidence suggests that some ENMs may exert adverse effects on the lung and other organ systems (Xia et al. 2009). These potential risks must be addressed in order to develop safe nanotechnology, but setting and implementing exposure standards require predictable results proven reliable by repeatability and agreement among multiple investigators. Because of the lack of standard protocols and reagents, ENM toxicity studies are difficult to compare because of inconsistencies in health outcomes and/or toxic thresholds. Much of this discordance can be attributed to *a*) heterogeneity of ENMs from batch to batch; *b*) inherent difficulties of interlaboratory comparisons; *c*) agglomeration of particles, which often changes toxicity; *d*) method and duration of dosing and dose level; and *e*) method of ENM manipulation prior to testing.

In addition to dose, many factors influence the toxicity of ENMs, including surface characteristics, charge, and shape. Size alone is a major determinant because many bulk materials that are relatively inert become toxic when produced at the nanoscale (Borm et al. 2006; Nel et al. 2006). Commonly produced and high production volume ENMs are carbon-based (e.g., nanotubes, graphene, fullerenes), metal-based [e.g., gold, silver, quantum dots, titanium dioxide (TiO_2_), zinc oxide], or of a biologic nature (e.g., liposomes and viruses designed for gene or drug delivery) (Card et al. 2008). Which ENMs will present the greatest potential threat to human health depends on relative toxicity and on the potential for exposure. TiO_2_ is one of the most widely used nanoscale materials to date, and the conversion of bulk to nanoscale TiO_2_ in consumer products (e.g., sunscreens) and industrial products (e.g., paints) is rapidly increasing (Robichaud et al. 2009). From a human health standpoint, this is significant because bulk TiO_2_ nanoparticles demonstrate increased toxicity compared with larger TiO_2_ particles (Oberdörster et al. 2005). Moreover, TiO_2_ nanoparticles can be manipulated into wire and belt shapes. The production of multiwalled carbon nanotubes (MWCNTs) is also increasing rapidly, along with a diversity of manipulations to alter physical and chemical attributes for various applications in industry, electronics, and medicine. Several studies have already shown that some types of MWCNTs delivered to the lungs by inhalation, intratracheal instillation (IT), or oropharyngeal aspiration (OPA) cause inflammation and fibrosis (Bonner 2010).

The goal of the Nano GO Consortium, an interlaboratory, multiinvestigator project, was to determine whether independent investigators involved in National Institute of Environmental Health Sciences (NIEHS)-funded consortium studies could generate consistent data sets in rodents using a well-characterized and commonly sourced panel of ENMs (Xia et al. 2013) and harmonized protocols for nanoparticle dispersion, delivery to the lungs, and collection of tissues. ENMs tested by this consortium included TiO_2_ anatase/rutile nanospheres (TiO_2_-P25), 100% anatase spheres (TiO_2_-A), and anatase nanobelts (TiO_2_-NBs), as well as three different MWCNTs, including the original material (O-MWCNT), a purified form with partial metal removal (P-MWCNT), and a carboxylic acid–functionalized form (F-MWCNT). Four laboratories evaluated lung responses in C57BL/6 mice exposed to ENMs by OPA exposure, and three labs tested responses in Sprague-Dawley (SD) or Fischer 344 (F344) rats following IT exposure. The results presented here demonstrate that a standard protocol can be used across multiple labs to yield similar results in the pulmonary inflammatory response. We also make recommendations for future directions in testing ENMs in a harmonized fashion among multiple labs. These recommendations should serve to guide regulatory agencies in making decisions regarding standard setting for occupational and environmental exposures to ENMs that present potential risks to human health and the environment.

## Materials and Methods

*ENMs.* The physical and chemical characteristics of nanoparticles used in this study are described in detail by Xia et al. (2013). We used three different formulations of TiO_2_ nanoparticles: TiO_2_-P25, TiO_2_-A, and TiO_2_-NBs. We obtained the TiO_2_-A from P. Biswas (Department of Energy, Environmental & Chemical Engineering, Washington University, St. Louis, MO); TiO_2_-P25 nanoparticles from Evonik (Essen, Germany); TiO_2_-NBs from N. Wu (Mechanical and Aerospace Engineering, West Virginia University, Morgantown, WV); MWCNTs from S. Mitra (Department of Chemistry and Environmental Science, New Jersey Institute of Technology, Newark, NJ); O-MWCNT from CheapTubes, Inc. (Brattleboro, VT). Two modified forms of O-MWCNTs were tested: P-MWCNT was derived from O-MWCNT by acid purification to remove residual metal catalyst, and F-MWCNT was derived from O-MWCNT by the addition of –COOH groups via carboxylic acid treatment to the nanotube surface.

*Preparation of ENM suspensions.* ENMs were suspended in dispersion medium (DM) containing disaturated phosphatidylcholine in 100% ethanol (DSPC; Sigma-Aldrich, St. Louis, MO); rat, mouse, or bovine serum albumin (Sigma-Aldrich); and 0.9% sterile saline (Porter et al. 2008). Spherical TiO_2_ nanoparticle and MWCNT suspensions were dispersed using a cup-horn sonicator (three mouse labs) for 1 min or a probe sonicator (rat labs and one mouse lab) for 30 min using a 10-sec on/off duty cycle and ice bath to disperse the particles and ensure that the sample temperature did not exceed 28°C. TiO_2_-NBs were not sonicated because preliminary studies showed that this causes axial fractures. Instead, TiO_2_-NBs were suspended in DM using gentle mechanical stirring for 60 min at room temperature. Control animals received DM alone that had been sonicated/stirred as described above.

*Consortium labs.* Four labs evaluated the lung responses of C57BL/6 mice exposed to ENMs by OPA exposure, and three labs tested responses in SD or F344 rats using IT exposure. There were four mouse lab groups: North Carolina State University (ML1), East Carolina University (ML2), Michigan State University (ML3), and University of Washington (ML4). There were three rat lab groups: National Institute for Occupational Safety and Health (RL1; SD rats), University of California, Davis (RL2; SD rats), and University of Rochester (RL3; F344 and SD rats). Lab codes were randomly assigned, and no identification is implied here. The first round of *in vivo* studies involved three independent labs, all of which used SD rats that were exposed to either TiO_2_-P25 or TiO_2_-A in DM. None of the treatment groups reported statistically significant changes in lung inflammatory parameters relative to DM controls; therefore, the scope of the consortium studies was expanded to include another rat strain (F344) and another species (mouse, C57BL6). The goal of this expansion was to strengthen conclusions about the relative toxicity of the selected ENMs that were evaluated.

*Experimental design.* ENMs (10, 20, or 40 µg in 50 µL DM) were delivered to the lungs of 6- to 8-week-old male C57BL/6 mice (20–25 g body weight) via OPA. TiO_2_ particles (20, 70, or 200 µg in 250 µL DM) or MWCNTs (10, 50, or 200 µg in 250 µL DM) were delivered to the lungs of 8- to 10-week-old male SD and F344 rats (350–420 g body weight) via IT. Lung tissues and bronchoalveolar lavage fluid (BALF) were collected at 1 and 7 days after exposure to TiO_2_ nanoparticles and 1 and 21 days after exposure to MWCNTs. BALF samples (three per mouse and five per rat) for each animal were combined and evaluated for total and differential cell counts [macrophages, neutrophils, eosinophils, and lymphocytes] and for measurement of total protein concentration and lactate dehydrogenase (LDH) activity. Total cell numbers were determined by counting the cells on three nonoverlapping images taken at 20× magnification, and the percentage of cell type per total cell population was determined by counting 500 cells per sample. For histopathological analysis, tissue sections from the left lung lobe were stained with hematoxylin and eosin (H&E). Immunohistochemistry was performed on tissue sections using a monoclonal rat anti-mouse neutrophil (allotypic marker clone 7/4) antibody (AbD Serotec, Raleigh, NC). Additional details are provided in Supplemental Material, Supplemental Methods and Materials, pp. 3–4 (http://dx.doi.org/10.1289/ehp.1205693). We present values as the percentage of neutrophils because it is not possible to compare effects of ENMs across labs or species using absolute neutrophil numbers as a result of variability related to lung size and lavage technique. All animals were treated humanely and with regard for alleviation of suffering.

*Statistical analysis.* Data are presented as mean ± SE for groups of four to six mice or rats. We performed two-way analyses of variance (ANOVA) and post hoc Tukey’s test or post hoc Bonferroni *t*-test. The analyses considered the main effects of and interactions between the factors, dose, and lab. In many cases, the two-way interaction was not statistically significant, so independent one-way analyses were performed. We considered *p* ≤ 0.05 to be statistically significant. Statistical analyses were performed using Graphpad Prism 5 (Graphpad Software, LaJolla, CA) and SigmaPlot 11 (Systat Software Inc., San Jose, CA).

## Results

**Figure 1:**
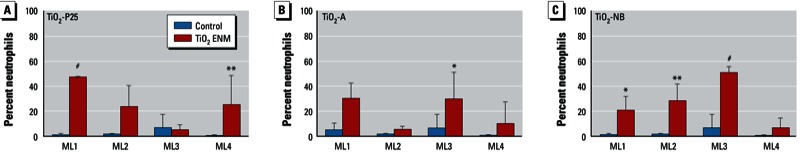
Stimulation of neutrophilic inflammation in the lungs of C57BL/6 mice by TiO_2_‑P25 (*A*), TiO_2_-A (*B*), or TiO_2_‑NB (*C*) evaluated in four labs (ML1, ML2, ML3, and ML4). Values are the mean ± SE percentage of neutro­phils in BALF 1 day after exposure to DM only (control) or TiO_2_ ENMs at 40 µg/50 µL. *p < 0.05, **p < 0.01, and ^#^*p* < 0.001 compared with controls.

**Figure 2:**
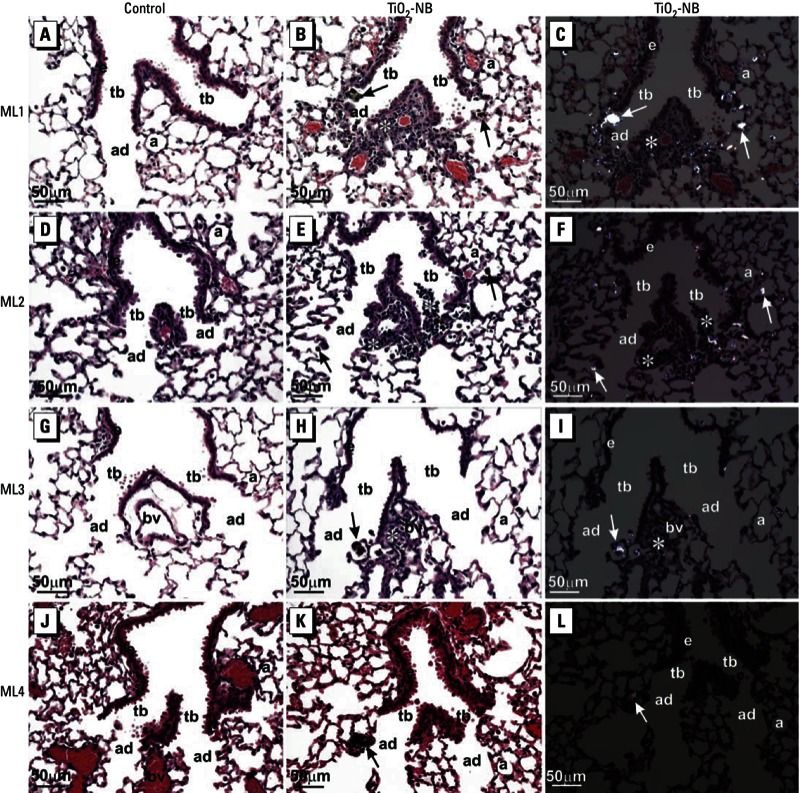
Photomicrographs of H&E-stained lung tissue 1 day after exposure of C57BL/6 mice to DM alone (control; *A*, *D*, *G*, *J*) or TiO_2_‑NB at 40 μg/50 µL DM (*B*, *C*, *E*, *F*, *H*, *I*, *K*, *L*). Photomicrographs are representative sections from each of the four labs: (*A–C)* ML1, (*D–F*) ML2, (*G–I*) ML3, and (*J–L*) ML4. (*A*, *D*, *G*, *J*, *B*, *E*, *H*, *K*) Bright-field light microscopy of representative sections of lung from control mice (*A*, *D*, *G*, *J*) and TiO_2_‑NB–exposed mice (*B*, *E*, *H*, *K*); sections from TiO_2_‑NB–exposed mice show inflammatory lesions primarily localized to alveolar duct bifurcations. (*C*, *F*, *I*, *L*) Polarized light microscopy of the same images shown in *B*, *E*, *H*, and *K* show inflammation at an alveolar duct bifurcation. Abbreviations: a, alveolus; ad, alveolar ducts; bv, blood vessel; e, airway epithelium; tb, terminal bronchioles. Arrows indicate TiO_2_‑NBs within macrophages at alveolar duct bifurcations, and asterisks (*) indicate inflammatory foci. All photo­micrographs were taken at the same magnification; bar = 50 μm.

*Mice.* TiO_2_. Differential counting of cells retrieved in BALF showed that all TiO_2_ ENMs tested increased the percentage of neutrophils relative to other cell types (macrophages, lymphocytes, and eosinophils). Results are shown only for the highest dose (40 μg/50 μL) because no significant effect on lung inflammation was observed at the lower doses. At 1 day postexposure, the relative percentage of neutrophils was significantly increased by TiO_2_-P25 in two of four labs (Figure 1A), by TiO_2_-A in one of four labs (Figure 1B), and by TiO_2_-NB in three of four labs (Figure 1C). By day 7 postexposure, neutrophilic inflammation in all TiO_2_ treatment groups returned to nearly baseline levels. Macrophages comprised > 95% of total BALF cells retrieved from the lungs of control mice (exposed to DM alone) (data not shown). TiO_2_ nanoparticles did not cause significant increases in the relative percentages of lymphocytes or eosinophils, except for a slight increase in the relative percentage of eosinophils observed by one lab after treatment with TiO_2_-NB (data not shown). BALF cytospins confirmed that inflammation caused by nanoparticle exposure was due primarily to neutrophil influx (data not shown). All four mouse labs observed that TiO_2_-NB caused an inflammatory response at the terminal bronchiolar region and the alveolar duct bifurcation region of the distal lung in mice 1 day after OPA delivery (Figure 2). TiO_2_-NBs were easily detectable by polarized light microscopy and were localized primarily in alveolar macrophages.

**Figure 3:**
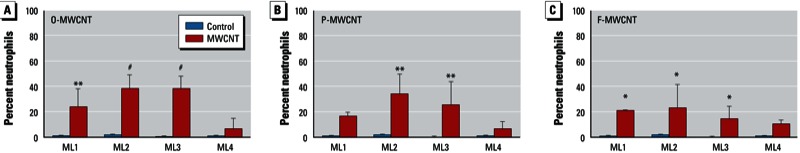
Stimulation of neutrophilic inflammation in the lungs of mice by O-MWCNT (*A*), P-MWCNT (*B*), or F-MWCNT (*C*) evaluated in four labs (ML1, ML2, ML3, ML4). Values shown are the mean ± SE percentage of neutro­phils in BALF 1 day after exposure to DM only (control) or MWCNTs at 40 µg/50 µL. (*A*) Significant increase induced by O-MWCNT in all four labs. (*B*) Significant increase induced by P-MWCNT in two labs. (*C*) Significant increase induced by F-MWCNT in three labs, F-MWCNT albeit to a lesser extent than that induced by O-MWCNT. *p < 0.05, **p < 0.01, and ^#^*p* < 0.001 compared with controls.

MWCNTs. Differential cell counts showed that the percentages of neutrophils were increased relative to other cell types in mouse BALF at 1 day postexposure to all forms of MWCNTs (O-MWCNT, P-MWCNT, and F-MWCNT) at 40 μg/50 μL (Figure 3). Lower doses of MWCNTs caused no significant effects on neutrophilia. In mice treated with MWCNTs, no significant increases were observed in the relative percentages of lymphocytes or eosinophils (data not shown). Light microscopic images of BAL cytospins confirmed that changes in the relative percentage of BAL cells were due to a significant increase in neutrophils produced by treatment with MWCNTs (data not shown). O-MWCNTs caused the greatest increases in neutrophils at 1 day postexposure in three of four labs compared with P-MWCNTs and F-MWCNTs (Figure 3). The inflammatory responses to all MWCNTs subsided to control levels by 21 days postexposure (data not shown). Combining the data from the four labs demonstrated the following order of potency: O-MWCNTs > P-MWCNTs > F-MWCNTs, albeit not to a significant extent [see Supplemental Material, Figure S1 (http://dx.doi.org/10.1289/ehp.1205693)]. However, combining the data from three of the four labs (ML1, ML2, and ML3) that initially showed significant effects of MWCNTs on neutrophils demonstrated a significant difference between O-MWCNTs and F-MWCNTs (see Supplemental Material, Figure S1). All four labs demonstrated that total protein and LDH levels in BALF were increased by all forms of MWCNTs or TiO_2_ nanoparticles, although these two end points of lung injury were highly variable among labs (data not shown).

**Figure 4:**
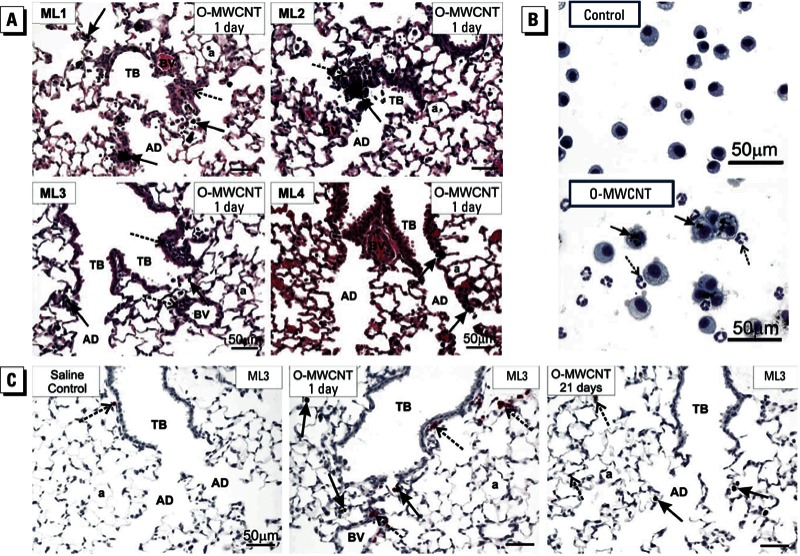
Results of lung histopathology (*A*), BAL cytospins (*B*), and immunohistochemistry (*C*) for mice exposed to DM only (control) or O-MWCNTs (40 μg/50 μL). Abbreviations: a, alveolus; AD, alveolar ducts; BV, blood vessel; TB, terminal bronchioles. (*A*) Histopathology showing lung inflammatory response to O-MWCNTs. Centriacinar bronchiolitis/alveolitis (dashed arrows) was induced by O-MWCNTs in three of four labs (ML1, ML2, ML3); one lab (ML4) found some deposition of O-MWCNTs in alveolar ducts with marginal inflammation. Solid arrows indicate macrophages containing O-MWCNT. (*B*) BAL cytospin images of cells from the lungs of mice exposed to DM (control) or O-MWCNTs; > 95% of macrophages from O-MWCNT–exposed animals were enlarged, activated alveolar macro­phages with numerous MWCNT inclusions (solid arrows) and neutrophils that do not contain MWCNTs (dashed arrows). The images are from a single lab (ML3) but are typical of responses from ML1 and ML2. (*C*) Immunohistochemistry using a monoclonal rat anti-mouse neutrophil (allotypic marker clone 7/4) antibody showing the location of neutro­phils (dashed arrows) near terminal bronchioles and in relation to macrophages containing O-MWCNT (solid arrows). Representative images are from ML3.

Histopathological analysis was used to determine the site of deposition of MWCNTs and the type of inflammatory lesions caused by oropharyngeal exposure to MWCNTs. Figure 4A shows that O-MWCNTs delivered by OPA caused centriacinar bronchiolitis/alveolitis in mice in three of four labs (ML1, ML2, and ML3). One lab (ML4) observed O-MWCNTs primarily within alveolar ducts with little inflammation. In ML4, far fewer O-MWCNTs appeared to be delivered to the lungs (Figure 4A), which correlated with much lower percentages of neutrophils recovered from the mice in that lab (Figure 3A). The inflammatory response associated with MWCNT exposure was characterized by neutrophilia as determined by BALF cell differentials (Figure 4B) and by using immunohistochemical staining with an anti-neutrophil antibody, which revealed neutrophils near terminal bronchioles and in proximity to macrophages containing O-MWCNTs (Figure 4C).

**Figure 5:**
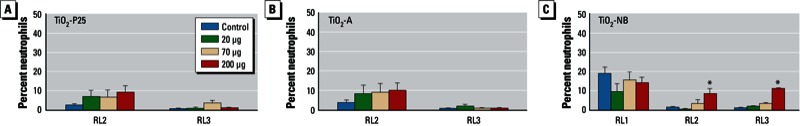
Stimulation of neutrophilic inflammation in the lungs of rats by TiO_2_‑P25 (*A*), TiO_2_-A (*B*), or TiO_2_‑NB (*C*) evaluated in three labs (RL1, RL2, and RL3). Values are the mean ± SE percentage of neutro­phils in BALF 1 day after exposure to DM only (control) or TiO_2_ ENMs (20, 70, or 200 µg/250 µL). *p < 0.05 compared with controls.

*Rats.* TiO_2_. Neither of the spherical TiO2 nanoparticles—TiO_2_-P25 nor TiO_2_-A—caused dose-related changes in the percentage of BALF neutrophils in rats in any of the labs, whereas TiO_2_-NBs caused significant neutrophilia in a dose-dependent manner in two of three labs (Figure 5A–C). However, there were visible TiO_2_ inclusions in macrophages recovered in BALF from exposed rats, indicating that TiO_2_ nanospheres reached the distal lung after IT. By day 7, all responses had returned to control values. No significant differences were observed between control (DM only) and TiO_2_-exposed animals with respect to total protein and LDH assays (data not shown).

**Figure 6:**
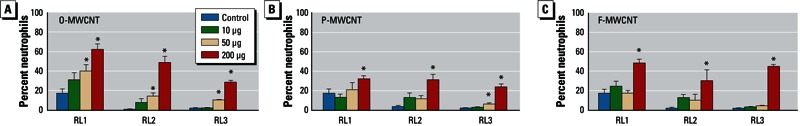
Stimulation of neutrophilic inflammation in the lungs of rats by O-MWCNT (*A*), P-MWCNT (*B*), or F-MWCNT (*C*) evaluated in three labs (RL1, RL2, and RL3). Values are the mean ± SE percentage of neutro­phils in BALF of rats 1 day after treatment with DM only (control) or MWCNTs (10, 50, or 200 µg/250 µL). *p < 0.05 compared with controls.

MWCNTs. Neutrophils were significantly elevated in rats exposed to the highest dose of all types of MWCNTs (200 μg/rat) compared with controls (DM alone) (Figure 6). All three rat labs (RL1, RL2, and RL3) found a significant dose-dependent effect of the MWCNTs at day 1 postexposure: The percentages of neutrophils were significantly elevated in the 50-µg/rat and 200-µg/rat dose groups compared with controls. Histological evaluation of lung tissue sections demonstrated the presence of inflammatory cells within centriacinar regions similar to that observed in mice (data not shown). Two of three labs also observed a significant effect on neutrophils at the highest dose of O-MWCNTs at 21 days postexposure (data not shown). Although all of the labs found a significant increase in the percentage of neutrophils for the highest dose of P-MWCNTs (200 µg/rat) at 1 day postexposure, the percentage was lower for P-MWCNT than for the same dose of O-MWCNT or F-MWCNT [see Supplemental Material, Figure S2 (http://dx.doi.org/10.1289/ehp.1205693)]. Two of three labs observed a small but significant effect in the high-dose P-MWCNT group at 21 days postexposure. The percentage of neutrophils in BALF of rats exposed to F-MWCNTs was significantly elevated at the highest dose in all labs at day 1 postexposure, but the neutrophilia was reduced compared with O-MWCNTs at 1 day postexposure and with O-MWCNTs and P-MWCNTs at 21 days postexposure. Interlaboratory precision was high enough to combine results from labs. This permitted a MWCNT toxicity ranking [see Supplemental Material, Figure S2 (http://dx.doi.org/10.1289/ehp.1205693)], which showed that at the 200-μg dose at 1 day postexposure, O-MWCNTs and F-MWCNTs caused stronger neutrophilic influx than the P-MWCNTs. At 21 days postexposure, there was no significant difference between rats that received DM (control) or the highest dose of F-MWCNTs (see Supplemental Material, Figure S2). However, significant neutrophilia persisted at 21 days postexposure for rats exposed to the highest dose of O-MWCNTs and P-MWCNTs. No significant differences were observed between control and MWCNT-exposed rats with respect to total protein and LDH assays (data not shown). Histopathological examination of the lungs demonstrated acute inflammatory lesions at 1 day postexposure, but no lasting changes were present at 21 days postexposure (data not shown).

## Discussion

The results presented here represent the first attempt by an integrated consortium of independent labs to determine whether the effects of well-characterized ENMs on pulmonary responses in mice and rats could be reliably reproduced by multiple investigators using a harmonized protocol. ENMs presented a challenge for interlaboratory comparisons because of the complexity of variables, including dispersion and characterization. Despite variability between labs in end points such as cell counts and LDH and total protein in BALF, other end points revealed consistent patterns among labs and between rodent models, including neutrophilic inflammation (as a percentage of total cells), and pathologic responses in mice treated with TiO_2_-NBs or O-MWCNTs. Because inhalation is a primary route of exposure to particles, the lungs represent a major target organ for the toxicity of ENMs from occupational, accidental, or environmental exposures (Kreyling et al. 2010). In the present study, all of the labs used high bolus doses of ENMs that were delivered by either OPA in mice or IT in rats. This approach was not intended to mimic real-world exposure conditions or deposition patterns in the lung that would occur via inhalation. Instead, we sought to evaluate whether different labs could generate similar and reproducible results with ENMs. Further work should focus on results after inhalation exposure, which is more physiologically relevant, to develop data for risk assessment and characterization to derive exposure standards.

The primary end point that was reliably reproducible in this interlaboratory effort was acute neutrophilia. However, the four mouse labs found different orders of potency for the three types of TiO_2_ nanoparticles. For example, for day 1, ML1 showed that TiO_2_-P25 produced the greatest level of neutrophilia, whereas ML3 showed that TiO_2_-NB caused the highest level of neutrophilia. TiO_2_-NBs were generally more toxic than TiO_2_ nanospheres—perhaps because of their shape—and produced neutrophilia in nearly all labs. However, neutrophilia caused by TiO_2_ nanoparticles did not persist at 7 days postexposure. Therefore, although acute inflammatory end points may not be the most useful for determining chronic disease such as fibrosis or carcinogenesis, acute inflammation is still the most sensitive end point for toxicity ranking. Future studies comparing spherical to high-aspect-ratio TiO_2_ should *a*) address clearance versus retention in the lungs after inhalation, *b*) evaluate pathologic changes over a period of weeks to months, and *c*) measure appropriate biomarkers of fibroproliferative and neoplastic disease.

All of the labs observed similar acute inflammatory responses to MWCNTs in mice and rats, as indicated by significant neutrophilic influx into the lungs of exposed animals and similar lung pathology. Three of four mouse labs found O-MWCNTs to be the most inflammatory, as indicated by neutrophil influx at 1 day postexposure, whereas F-MWCNTs were the least proinflammatory. All three rat labs also observed that O-MWCNTs were the most inflammatory [see Supplemental Material, Figure S2 (http://dx.doi.org/10.1289/ehp.1205693)]. This judgment about relative toxicological potency is based on the consistent findings of an onset of inflammation at a lower dose (i.e., 50 μg IT) combined with persistence of neutrophilia at 21 days postexposure. Therefore, both mice and rats used in the consortium effort independently identified O-MWCNTs as having the greatest proinflammatory effect, suggesting that residual catalytic metals [see Xia et al. (2013) for physicochemical characterization] may have contributed to the inflammatory response. Moreover, the mouse and rat working groups observed similar pathology in animals exposed to O-MWCNTs.

In retrospect, we can identify aspects of the study design that worked well and those that could be considered for improvement in future interlaboratory *in vivo* comparison studies with ENMs. The concordance in findings in terms of neutrophilia and histopathological changes that were detected with the TiO_2_-NBs and MWCNTs confirms the importance of careful planning and lends strength to conclusions made about the relative acute potency of these materials. However, the variability found in the absolute number of neutrophils highlights some methodological differences that were not considered at the outset of the studies. Even though by design the rat and mouse groups used similar dispersion protocols to break up large agglomerates, it was beyond the scope of this project to ensure that all users had identical dispersion instrumentation. Thus, it was not technically possible to ensure that all labs delivered ENMs that were identical in particle size distribution. Approaches that have been recently described (Taurozzi et al. 2011, 2013) could be considered for future efforts, but they were not available at the time the studies were conducted.

Other design problems to overcome include differences in instillation/aspiration and lavage techniques from one lab to another. Despite detailed planning, it was not realistic to mandate identical techniques across the labs because that would have involved significant expenditure of animals and time for training. It may not be possible to overcome these problems; thus, careful consideration should be given to the end points that can be reliably used for comparisons. Last, inherently low-toxicity ENMs such as TiO_2_ are perhaps not as useful for interlaboratory comparisons as more potent materials. More specifically, the spherical TiO_2_ particles were not potent enough in these bioassays to be identified consistently across the labs as having acute *in vivo* toxicity upon bolus delivery. In contrast, the MWCNTs were sufficiently potent to be identified as potentially hazardous by all labs in this round-robin effort (i.e., sufficiently insensitive to the methodological differences among the labs).

It is important to note that pathology might be affected by nanoparticle delivery modes. The fibrotic response to MWCNTs in mice has been reported to be more diffuse in inhalation exposures compared with aspiration exposure (Shvedova et al. 2008). The issue of MWCNT dispersion (i.e., agglomeration state) and consequent disease is a critical one in assessing health risks. For example, inhalation of agglomerated MWCNTs causes a different pathology (less interstitial fibrosis) than dispersed fibrillar structures (Ma-Hock et al. 2009; Pauluhn 2010). Other recent studies (Porter et al. 2010; Wang et al. 2011) reported that OPA aspiration of well-dispersed MWCNTs caused more fibrosis and growth-factor production than did nondispersed MWCNTs. All of the labs in the consortium used the same dispersion medium for suspending MWCNTs or TiO_2_ nanoparticles. Nevertheless, it was apparent that some agglomeration occurred, particularly for MWCNTs. Future studies should carefully evaluate whether functionalization of MWCNTs or other ENMs influences agglomeration status and surface properties, which could in turn alter pathologic responses resulting from bolus delivery of particles to the lungs in a liquid suspension. Responses following more realistic and physiological inhalation exposures should be evaluated with these materials to confirm the hazard ranking and to derive the lowest or no-effect levels for purposes of risk characterization, although that was not the purpose of the experiments presented here.

MWCNTs and TiO_2_-NBs represent ENMs that are referred to as high-aspect-ratio nanoparticles (i.e., fiber-like structures that have nanoscale width but can be micrometers in length). A principal characteristic of high-aspect-ratio nanoparticles that is shared by pathogenic fibers, such as asbestos, is impeded clearance from the lungs after inhalation exposure, which may lead to the pathogenesis of diseases such as pulmonary fibrosis and mesothelioma (Bonner 2010). There is evidence that high-aspect-ratio TiO_2_-NBs and long MWCNTs are more pathogenic and may elicit “frustrated phagocytosis” by macrophages, lysosomal disruption, and impaired clearance (Donaldson et al. 2011; Hamilton et al. 2009). Therefore, exposure to some of the ENMs used in this consortium study might be cause for concern about human health.

Pulmonary toxicology studies in rodents have shown that OPA or IT exposure to MWCNTs at high doses/concentrations results in lung inflammation and interstitial pulmonary fibrosis, similar to that of exposure to asbestos fibers (Han et al. 2010; Mercer et al. 2011; Porter et al. 2010; Ryman-Rasmussen et al. 2009a; Wang et al. 2011). Inhaled MWCNTs can also cause inflammatory reactions by migrating to the pleural membrane surrounding the lungs (Porter et al. 2010; Ryman-Rasmussen et al 2009b). Direct intraperitoneal injection of very high doses of other MWCNTs have also been reported to induce mesothelioma in genetically susceptible mice that lack an allele of the tumor suppressor p53 (Takagi et al. 2008). Some caution should be taken in interpreting these latter results, because p53-deficient mice spontaneously develop tumors. In addition, a 2-year study showed that MWCNTs possessed no carcinogenicity when injected into the peritoneal cavity of male Wistar rats (Muller et al. 2009), although that study used very short MWCNTs (< 1 μm). However, the injection studies mentioned above did not address direct exposure to the lungs. It should also be stressed that it is unclear to what extent the differences in MWCNT properties (i.e., associated metal content, dispersion state/method, aspect ratio, rigidity) and means of exposure played a role in disparate outcomes. Therefore, it remains to be determined whether MWCNTs are carcinogenic in inhalation studies.

Thus far, no human disease has been linked to exposure to carbon nanotubes. Therefore, the scientific community has a unique opportunity to address human health effects in a preventive manner and reduce potential adverse health effects by establishing assays to predict disease outcome using *in vitro* and *in vivo* models. This article on interlaboratory studies in rodents exposed to ENMs and the accompanying article on interlaboratory *in vitro* studies (Xia et al. 2013) comprise one of the first attempts to assess the reproducibility of experiments among different labs using harmonized experimental protocols and identical ENMs.

## Outlook and Future Directions

As part of the nanotechnology revolution, a vast array of industrial and consumer products is emerging on the market. Many nanomaterials are already in the marketplace in consumer products [a comprehensive list is maintained and updated by the Project on Emerging Technologies (2013)]. It is not feasible or practical to test all of these ENMs using *in vivo* rodent models. Therefore, predictive high throughput screening of materials using validated *in vitro* model systems, along with with more thorough analyses of selected ENMs using realistic *in vivo* rodent models and realistic exposures, will likely be the only practical way to determine the toxic potential of an enormous variety of emerging nanomaterials (Nel et al. 2009). Standard protocols should be used for *in vitro* screening and the resulting data made publicly available through a centralized database. In addition, a centralized repository for ENMs should be established to provide well-characterized ENMs in large quantities to investigators.

We are only beginning to understand the mechanisms of toxicity of the increasing variety of emerging ENMs. Growing evidence indicates that nanosizing particles increases toxicity because of a proportional increase in surface area that is then available to generate reactive oxygen species (ROS), primary factors that drive cellular stress and disease pathogenesis (Nel et al. 2006). However, in addition to the obvious role of increased surface area and ROS generation, nanosized particles could be capable of moving across cellular barriers to interact with subcellular structures (e.g., mitochondria, microtubules, organelles, DNA) in potentially unique ways that we do not yet fully comprehend. There is also evidence that ENMs traverse the pulmonary epithelial–endothelial barrier to gain access to secondary target tissues (Aiso et al. 2011; Kreyling et al. 2002, 2009). Alternatively, ENMs could influence immune responses of secondary target tissues by stimulating the release of lung cytokines into the systemic circulation (Mitchell et al. 2007). Therefore, to keep pace with the emerging nanotechnology industry and associated human and environmental exposures, more research is required to elucidate common mechanisms of nanoparticle-induced adverse health effects.

Unfortunately, the amount of funding for nanotoxicology research on health effects lags far behind what is available for nanotechnology research and development. The disproportionate emphasis on nanotechnology research and development compared with issues of risk for human health and the environment could result in a new wave of occupational and environmental health crises. Although many ENMs will present little or no risk, it is inevitable that at least some ENMs will pose a significant risk to human health and the environment. Our consortium experiments described here are significant in providing results of a coordinated effort toward addressing hazard identification of high-priority ENMs. Future research should expand this consortium effort to allow for toxicity testing and exposure assessment to ensure the safe continuation and economic viability of nanotechnology.

## Supplemental Material

(606 KB) PDF
