# Supplementary material for: Interlaboratory Evaluation of Rodent Pulmonary Responses to Engineered Nanomaterials: The NIEHS Nano GO Consortium
Source: Environ Health Perspect. 2013 May 6;121(6):676–82. doi: 10.1289/ehp.1205693 (PMC3672912; doi:10.1289/ehp.1205693)
Supplement: (606 KB) PDF [file ehp.1205693.s001.pdf]

Environmental Health Perspectives doi:10.1289/ehp.1205693

Supplemental Material

Interlaboratory Evaluation of Rodent Pulmonary Responses to Engineered Nanomaterials: The NIEHS NanoGo Consortium

James C. Bonner, Rona M. Silva, Alexia J. Taylor, Jared M. Brown, Susana C. Hilderbrand, Vincent Castranova, Dale Porter, Alison Elder, Günter Oberdörster, Jack R. Harkema, Lori A. Bramble, Terrance J. Kavanagh, Dianne Botta, Andre Nel, and Kent E. Pinkerton

Table of Contents

Supplemental Methods and Materials--- 3

Supplemental Material, Figure S1. Comparison of the lung inflammatory response in mice among four laboratories to the three forms of MWCNTs-------------------------------5

Supplemental Material, Figure S2. Comparison of the lung inflammatory response in rats among three laboratories to the three forms of MWCNTs-------------------------------6

Supplemental Methods and Materials

Mouse Working Group: Pathogen-free adult male C57BL6 mice 6 to 9 weeks of age (20 to 25 gm) were obtained from Charles River Laboratories (Raleigh, NC or Hollister, CA). Mice were housed in temperature and humidity controlled animal facilities with free access to food and water *ad libitum*. Animal care and use committee approval was obtained from all institutions for procedures involving mice in this study.

Lung Delivery of ENMs to Mice: ENMs were delivered to the lungs of 6-8 week old male C57BL/6 mice via OPA. An initial pilot experiment was performed wherein mice were exposed to a dose range of 10, 20, and 40 μg/50 μl (approximately equivalent to 0.5, 1, or 2 mg/kg) of TiO_2 nanoparticles or MWCNTs. Each animal was anesthetized with isoflurane and dosed with 10, 20, or 40 μg of TiO_2 or MWCNTs in 50 μL DM or 50 μL DM alone using an oropharyngeal aspiration board with the animal at a 45 degree angle suspended in place by a loop of suture under the upper incisors. Care was taken to gently extend the tongue with padded forceps and deliver the suspension via pipette to the back of the pharynx to ensure particle delivery to the lungs.

Necropsy and Tissue Collection: Mice were euthanized with an intraperitoneal injection of pentobarbital (ML1, ML2, ML3), or by CO_2 narcosis followed by cervical dislocation (ML4), and lungs were serially lavaged three times with 0.5 ml DPBS. All three lavages were combined and two 100 μL samples were taken for LDH (CytoTox 96® Non-Radioactive Cytotoxicity Assay; Promega, Madison, WI) and differential cell counts. A Thermo Scientific Cytospin 4 (Thermo Electron Corporation, Waltham, MA), or a Cyto-Tek Model 4325 Cytocentrifuge (Miles Scientific, Elkhart, IN) was used to plate cells from the BAL fluid sample onto glass slides followed by fixation and staining with the Diff-Quik® Stain Set (Dade Behring Inc, Newark, DE). Total cell numbers were determined by counting the cells on three non-overlapping images taken at 20X magnification for each sample. The percentage of cell type per total cell population was determined by counting 500 cells per sample. After lung lavage, the left lung lobe was intratracheally infused with 10% neutral buffered formalin for 48 hr then transferred to

70% ethanol. The fixed lung tissue was then cross-sectionally cut into three portions, embedded in paraffin, and stained with hematoxylin and eosin.

Rat Working Group: Pathogen-free adult male Sprague Dawley (SD) or Fischer 344 (F344) rats 8 to 10 weeks of age (BW 350-420 gm) were obtained from Harlan Laboratories (Hayward, CA), Charles River Laboratories (Ann Arbor, MI), or Hilltop Laboratories (Scottdale, PA). Rats were housed in temperature and humidity controlled animal facilities with free access to food and water. Animal care and use committee approval was obtained from all institutions for all procedures involving rats in this study.

Lung Delivery of ENMs to Rats: ENMs were delivered to the lungs of rats via IT. Each animal was anesthetized with isoflurane and exposed to 20, 70, or 200 µg of TiO_2 nanoparticles or 10, 50, or 200 µg of MWCNT in 250 µL DM or 250 µL DM alone. Care was taken to deliver the instillate into the lungs during the inspiratory phase of the breathing cycle to improve particle distribution.

Necropsy and Tissue Collection: Rats were euthanized with an intraperitoneal injection of pentobarbital and bronchoalveolar lavage was performed using five separate 5 mL aliquots of 0.9% sterile saline. Aliquots one and two were kept separate from aliquots three through five. BALF was centrifuged at 4°C and 2000 rpm for 10 minutes. Supernatant from the first two lavages was collected for protein and LDH analyses, which were performed using kits from Thermo Scientific (Rockford, IL) and Sigma (St. Louis, MO). Cells from all lavages were combined and then resuspended in 0.9% sterile saline for determination of total cell numbers and cell viability using a hemacytometer and trypan blue exclusion. Cell differentials were determined by counting a minimum of 500 cells on cytospin slides that were stained with Diff-Quik®.

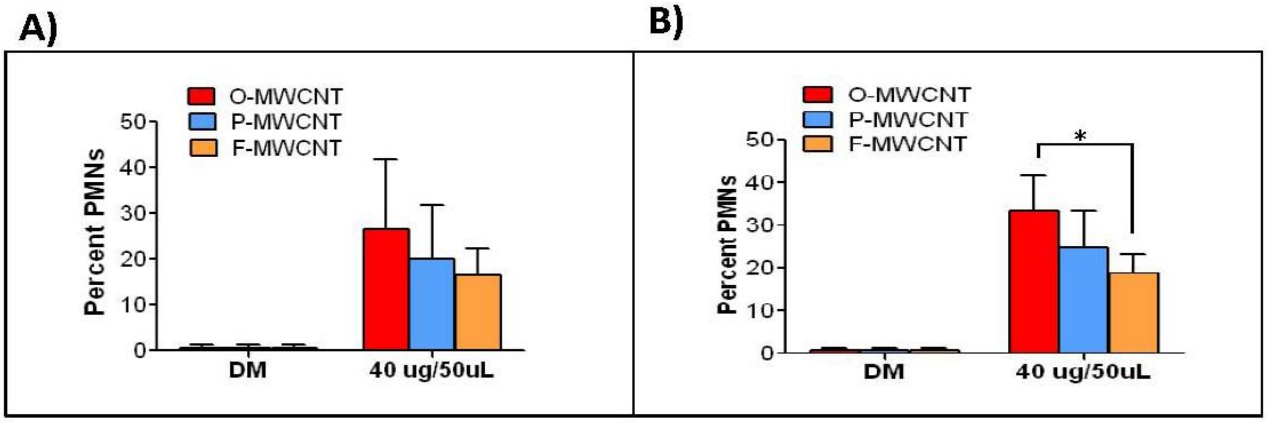

Supplemental Material, Figure S1. Comparison of the lung inflammatory response in mice among four laboratories to the three forms of MWCNTs (O-, P-, and F-MWCNT). Inflammation was indicated by increased neutrophils (i.e., polymorphonuclear cells, PMNs) at 1 day in BAL fluid. **A)** O-MWCNT consistently caused the greatest increase in PMNs but was not significantly different from P-MWCNT or F-MWCNT when grouping data from all four laboratories. **B)** The response to F-MWCNT was significantly less than the response to O-MWCNT (*P<0.05) when compared among three laboratories (ML1, ML2, ML3).

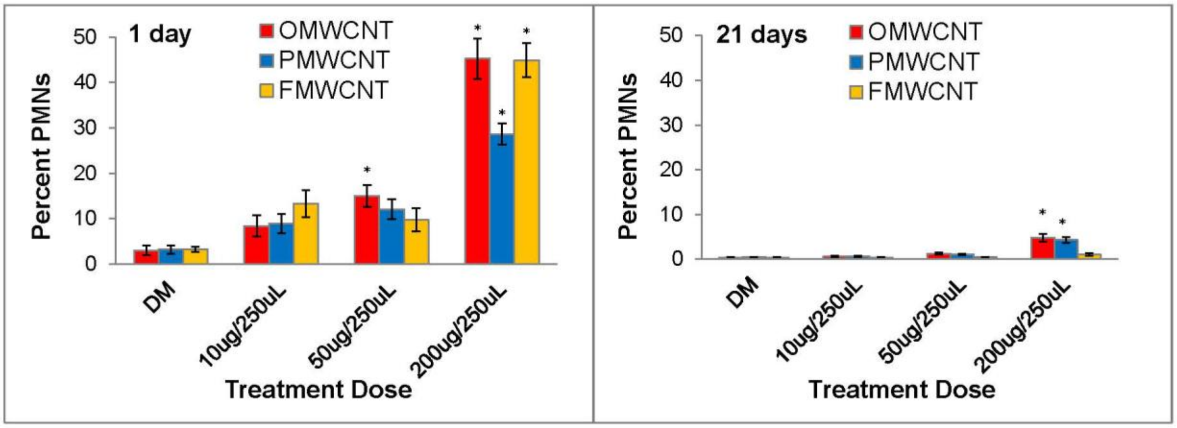

Supplemental Material, Figure S2. Comparison of the lung inflammatory response in rats among three laboratories (RL1, RL2, and RL3) to the three forms of MWCNTs (O-, P-, and F-MWCNT). The inflammatory response was indicated by increased neutrophils (i.e., polymorphonuclear cells, PMNs) in BAL fluid at 1 day (left panel) and 21 days (right panel). The multi-laboratory comparison showed that O-MWCNT and F-MWCNT caused the greatest increase in PMNs at 1 day and P-MWCNT caused lesser response. However, at 21 days, O-MWCNT and P-MWCNT showed persistent neutrophilia, whereas F-MWCNT did not cause a significant increase in PMNs. *P < 0.05.
